# Antimicrobial resistance and heterogeneity of *Neisseria gonorrhoeae* isolated from patients attending sexually transmitted infection clinics in Lusaka, Zambia

**DOI:** 10.1186/s12864-024-10155-y

**Published:** 2024-03-18

**Authors:** Kelvin Lutambo Sarenje, Marco van Zwetselaar, Happiness Kumburu, Tolbert Sonda, Blandina Mmbaga, Owen Ngalamika, Margaret C. Maimbolwa, Amon Siame, Sody Munsaka, Geoffrey Kwenda

**Affiliations:** 1 Department of Biomedical Sciences, School of Health Sciences, University of Zambia, Lusaka, P.O. Box 50110, Zambia; 2 Kilimanjaro Clinical Research Institute, Moshi, Kilimanjaro Tanzania; 3 Kilimanjaro Christian Medical Centre, Moshi, Tanzania; 4 Kilimanjaro Christian Medical University College, Moshi, Tanzania; 5 Department of Dermato-venereology, University Teaching Hospital, Lusaka, Zambia; 6 Department of Midwifery Child, and Women’s Health, School of Nursing Sciences, University of Zambia, Lusaka, Zambia; 7 Centre for Infectious Disease Research in Zambia, Lusaka, Zambia

**Keywords:** *Neisseria gonorrhoeae*, Molecular epidemiology, Whole-genome sequencing, Typing, Antimicrobial resistance, Zambia

## Abstract

**Background:**

Antimicrobial resistance (AMR) of *Neisseria gonorrhoeae* is a threat to public health as strains have developed resistance to antimicrobials available for the treatment of gonorrhea. Whole genome sequencing (WGS) can detect and predict antimicrobial resistance to enhance the control and prevention of gonorrhea. Data on the molecular epidemiology of *N. gonorrhoeae* is sparse in Zambia. This study aimed to determine the genetic diversity of *N. gonorrhoeae* isolated from patients attending sexually transmitted infection (STI) clinics in Lusaka, Zambia.

**Methods:**

A cross-sectional study that sequenced 38 *N. gonorrhoeae* isolated from 122 patients with gonorrhea from 2019 to 2020 was conducted. The AMR profiles were determined by the E-test, and the DNA was extracted using the NucliSens easyMaG magnetic device. Whole genome sequencing was performed on the Illumina NextSeq550 platform. The Bacterial analysis pipeline (BAP) that is readily available at: https://cge.cbs.dtu.dk/services/CGEpipeline-1.1 was used for the identification of the species, assembling the genome, multi-locus sequence typing (MLST), detection of plasmids and AMR genes. Phylogeny by single nucleotide polymorphisms (SNPs) was determined with the CCphylo dataset.

**Results:**

The most frequent STs with 18.4% of isolates each were ST^7363^, ST^1921^ and ST^1582^, followed by ST^1583^ (13%), novel ST^17026^ (7.9%), ST^1588^ (7.9%), ST^1596^ (5.3%), ST^11181^ (5.3%), ST^11750^ (2.6/%) and ST^11241^ (2.6%) among the 38 genotyped isolates. The *blaTeM-1B* and *tetM* (55%) was the most prevalent combination of AMR genes, followed by *blaTeM-1B* (18.4%), *tetM* (15.8%), and the combination of *blaTeM-1B*, *ermT*, and *tetL* was 2.6% of the isolates. The AMR phenotypes were predicted in ciprofloxacin, penicillin, tetracycline, azithromycin, and cefixime. The combination of mutations 23.7% was *gryA* (S91F), *parC* (E91G), *ponA* (L421) and *rpsJ* (V57M), followed by 18.4% in *gyrA* (S91F), *ponA* (L421P), *rpsJ* (V57M), and 18.4% in *gyrA* (D95G, S91F), *ponA* (L421P), and *rpsJ* (V57M). The combinations in *gyrA* (D95G, S91F) and rpsJ (V57M), and *gyrA* (D95G, S91F), *parC* (E91F), *ponA* (L421P) and *rpsJ* (V57M) were 13.2% each of the isolates. Plasmid *TEM-1* (84.2%), *tetM (*15.8%), and gonococcal genetic island (GGI) was detected in all isolates.

**Conclusion:**

This study revealed remarkable heterogeneity of *N. gonorrhoeae* with *bla*_TEM−1_, *tetM, ponA, gyrA*, and *parC* genes associated with high resistance to penicillin, tetracycline, and ciprofloxacin demanding revision of the standard treatment guidelines and improved antimicrobial stewardship in Zambia.

**Supplementary Information:**

The online version contains supplementary material available at 10.1186/s12864-024-10155-y.

## Background

The sexually transmitted infection (STI) gonorrhea caused by the bacterium *Neisseria gonorrhoeae* remains a major global public health concern because of its capacity to evolve high levels of resistance to antibiotics available for treatment [1, 2]. The superbug has developed plasmid-mediated and/or chromosomally mediated antimicrobial resistance (AMR) that has compromised the management of gonorrhea worldwide [3]. The AMR mechanisms are usually present in the gonococcal cell and/or a combination of genes with mutations within specific genes to cause resistance to antibiotics [4–6]. The World Health Organization (WHO) has declared *N. gonorrhoeae* as a priority pathogen because of its resistance to third-generation cephalosporins (3GS) and fluoroquinolones [7].

Whole genome sequencing (WGS) technology has allowed the tracking of transmission and prediction of AMR to control gonococcal infections [8–10]. Multi-locus sequence typing (MLST), *Neisseria gonorrhoeae* multi-antigen sequence typing (NG-MAST), and *Neisseria gonorrhoeae* sequence typing for antimicrobial resistance (NG-STAR) are different typing tools that have been used to study the molecular epidemiology of *N. gonorrhoeae* in terms of genetic lineages and clonal relationships to control the spread of drug-resistant genotypes [11–15]. MLST is based on the detection of sequence variation using seven conserved housekeeping genes, putative ABC transporter (*abcZ*), adenylate kinase (*adk*), shikimate dehydrogenase (*aroE*), furamase hydrase (*fumC*), glucose-6-phosphate dehydrogenase (*gdh*), pyruvate dehydrogenase subunit (*pdhC*), and phosphoglucomutase (*pgm*) [12]. The NG-MAST analyses are based on the variable internal fragments of highly polymorphic porin B (*porB*) and transferrin binding protein B (*tbpB*) [16]. The NG-STAR is based on AMR determinants (*penA, mtrR*, *porB1b*, *ponA*, *gyrA*, *parC*, and 23rRNA) [17]. However, the cost of WGS remains high and is not available in many parts of Africa [13, 18].

In 2020, the World Health Organization (WHO) estimated 82.4 million incident global cases of gonorrhea among adults 15–49 years of age [2]. The highest incidence rate of gonorrhea was found in sub-Africa with an increase of 0.2% in women and 1.1% in men every year [19, 20]. The development of effective vaccines and novel therapeutics would mitigate the emergence and spread of untreatable gonorrhea [21]. Gonorrhea can be concomitant with HIV and enhances its transmission [22–26].

The treatment of STIs is according to syndromic management guidelines which have contributed highly to the AMR due to empirical treatment in sub-Sahara Africa [27–30]. The Zambian standard treatment guidelines recommended the use of a single dose of ciprofloxacin in the treatment of gonorrhea (Ciprofloxacin 500 mg PO stat plus doxycycline 100 bd PO X 7/7) [31]. The WGS data that provides opportunities to understand the population structure of *N. gonorrhoeae* for prevention and control of gonorrhea was sparse despite AMR being an emerging phenomenon in Zambia [32].

This study aimed to determine the genetic diversity of *N. gonorrhoeae* isolated from patients attending STI clinics in urban hospitals in Lusaka, Zambia.

## Methods

### Study Design and Population

A cross-sectional study on 38 *Neisseria gonorrhoeae* isolated from 122 patients with gonorrhea attending STI clinics in urban hospitals in Lusaka, Zambia. The urethral and endocervical specimens were collected from patients who presented with a discharge from September 2019 to August 2020. The gonococcal isolates were submitted to the University Teaching Hospital (UTH) molecular laboratory for antimicrobial susceptibility testing (AST) and DNA extraction, and to Kilimanjaro Clinical Research Institute (KCRI) at the biotechnology laboratory (KCRI-BL) for molecular testing. The KCRI-BL is GCLP accredited, ISBN 978-1-904610-00-7 operated by Qualogy.

### Antimicrobial susceptibility testing

The minimum inhibitory concentrations (MICs; µg/mL) of the ciprofloxacin, ceftriaxone, spectinomycin, azithromycin, penicillin, and tetracycline were determined by the E-test (bioMerieux, Marcy-I’Etoile, France), on GC-chocolate with 1% Vitox supplement (Beckton Dickison, France) following the manufacturer’s instructions. The interpretation of MIC dilutions in susceptible (S), intermediate (I) and resistance (R) categories was according to Clinical and Laboratory Standard Institute (CLSI) criteria [33]. The plates were inoculated by dipping a sterile swab into a bacterial cell suspension adjusted to 0.5 McFarland standards using a turbidometer (Oxoid Integrated Technologies Ltd, England). The standardized inoculum was then streaked across the surface of the GC-chocolate agar. The plates were dried at ambient temperature for 5 min before applying the E-test strips and incubated at 36^o^C ± 1^o^C in 5% CO_2_ for 24 h. The SIR categories for antimicrobial agents in µg/mL were as follows: Ciprofloxacin (CIP) S; ≤0.06, I; 0.12–0.5, R; ≥1, ceftriaxone (CTX) S; ≤0.25, R; >0.25, spectinomycin (SPEC) S; ≤32, I; 64, *R* ≥ 128, cefixime (CFX) S; ≤0.25, R; >0.25, azithromycin (AZT) S; ≤1, R; >1, penicillin (PEN) S; ≤0.06, I; 0.12-1, R; ≥2, and tetracycline (TET) S; ≤0.25, I; 0.12-1, R; ≥2. *Neisseria gonorrhoeae* American Type Culture Collection (ATCC) 49,226 was used as a reference strain and was within the acceptable quality control ranges.

### Extraction of genomic DNA

DNA was isolated using the NucliSens easyMaG Nucleic Extraction platform (BioMerieux, Marcy-I’Etoile, France), according to the manufacturer’s instructions. A loopful of *Neisseria gonorrhoeae* from pure cultures grown on chocolate agar (Mast Diagnostics, Merseyside, UK) were transferred into a microcentrifuge tube containing 400µL of 1x TE buffer (10mM Tris-HCl [pH 8.0], 0.1mM EDTA [pH 8.0]) for the bacterial suspension. The bacterial preparation was transferred to the sample strip well of the extractor with the elution of 50µL. The DNA preparation was then preserved at -20^o^C before further analysis.

### DNA quantification and sequencing

The cgDNA was quantified using a Qubit V4.0 fluorometer (Invitrogen by Thermo Fisher Scientific). The volume of 2µL of DNA was quantified before sequencing. The whole genome sequencing (WGS) was performed using Illumina DNA library preparation protocol, document 1,000,000,025,416 v09 (Illumina Inc., San Diego, CA, USA). Input cgDNA of 100ng was used for library preparation. The amplified DNA library was cleaned using double-sided beads and purified in resuspension buffer (RSB). The paired-ends 150 bp indexed reads were generated using the mid-output protocol on the Illumina Nextseq550 platform (Illumina Inc., San Diego, CA, USA).

### Bioinformatics analyses

The raw sequence data was checked using FastQC 0.11.9 and screened for contamination using FastQScreen 0.15.1 against the Knead-Data human reference with decoy, and NCBI UniVec Core [34–36]. Untrimmed reads were processed by the KCRI-CGE Bacterial Analysis Pipeline (BAP) 3.6.5 using default settings [37, 38]. The BAP workflow was comprised of genome assembly with SKESA 2.4.0 and computation of assembly metrics with uf-stats 1.3.1 [39, 40]. Identification of *N. gonorrhoeae* species was performed with KmerFinder 3.0.2, and MLST typing with CGE MLST 2.0.9 and KCST 1.2.6 [41–46]. The AMR detection with ResFinder 4.2.3 and plasmid identification with PlasmidFinder 2.1.6 and pMLST 2.0.3 [47, 48] while the core genome MLST assignment was performed with cgMLSTFinder 1.1.5 [43, 45]. The assemblies were assessed for genome completeness and bacterial contamination with CheckM 1.2.2 and GUNC 1.0.5 + post1 [49, 50]. The Reference was made of 160 genomes for *Neisseria gonorrhoeae* with assembly level “Complete Genome” or “Chromosome” downloaded from NCBI RefSeq on 17 Oct 2022 and were annotated with their MLST using KCST 1.2.6 [14]. Pairwise average nucleotide identities (ANI) were computed between all study genomes and all reference genomes with FastANI 1.33 [51]. A phylogenetic tree was estimated from genome assemblies using SANS serif in ‘strict’ mode using default settings [52].

## Results

**Fig. 1:**
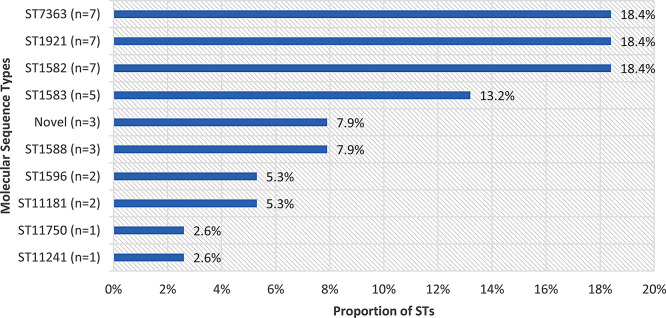
Frequency and percentage of sequence types of *N. gonorrhoeae*

A total of 38 isolates of *N. gonorrhoeae* were successfully sequenced and various sequence types (ST) were identified. The most frequent STs with 18.4% (7/38) of isolates each were ST^7363^, ST^1921^ and ST^1582^, followed by ST^1583^ (13%), novel ST^17026^ (7.9%), ST^1588^ (7.9%), ST^1596^ (5.3%) and ST^11181^ (5.3%). The ST^11750^ and ST^11241^ (2.6%) each had one representative (Fig. 1).

**Fig. 2:**
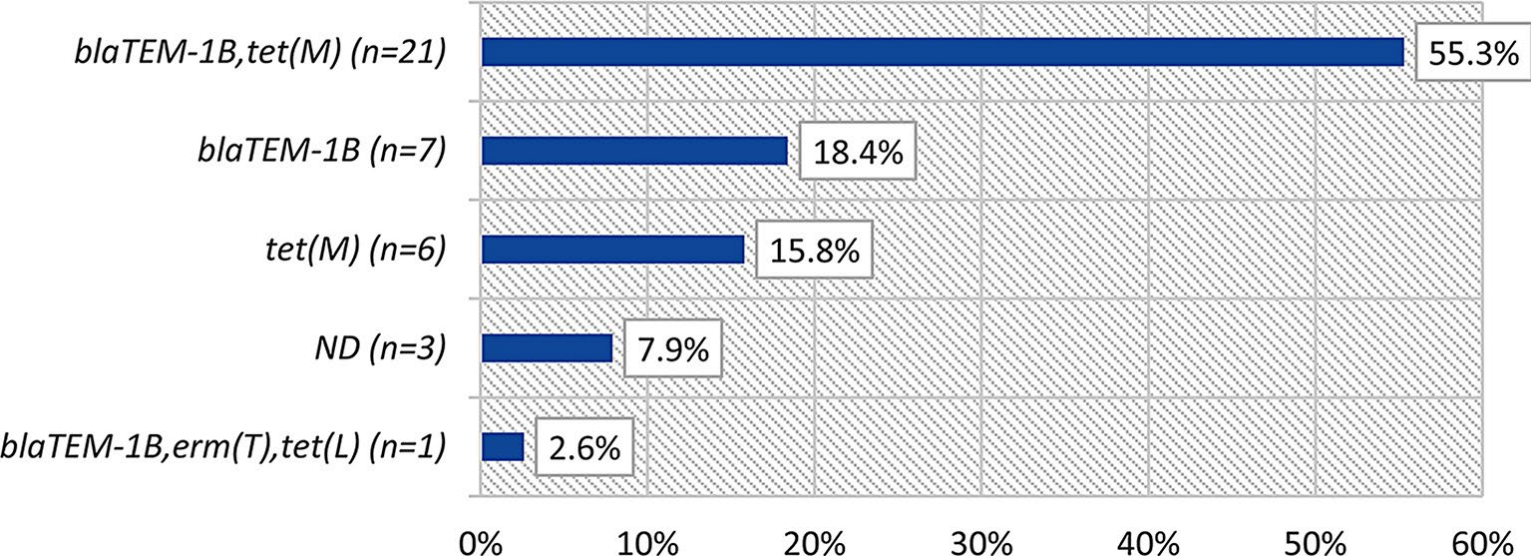
Frequency and percentage of AMR determinants of *N. gonorrhoeae*

The majority of isolates contained both *blaTEM-1B* and *tetM* (55%, 21/38), while 18.4% (7/38) contained *blaTEM-1B* only, 15.8% (6/38) contained *tetM* only, and one isolate contained *blaTEM-1B*, *ermT*, and *tetL* (Fig. 2). The Beta lactamase plasmid *TEM-1* was detected in 84.2% (32/38) and gonococcal genetic island (GGI) was detected in all isolates.

**Fig. 3:**
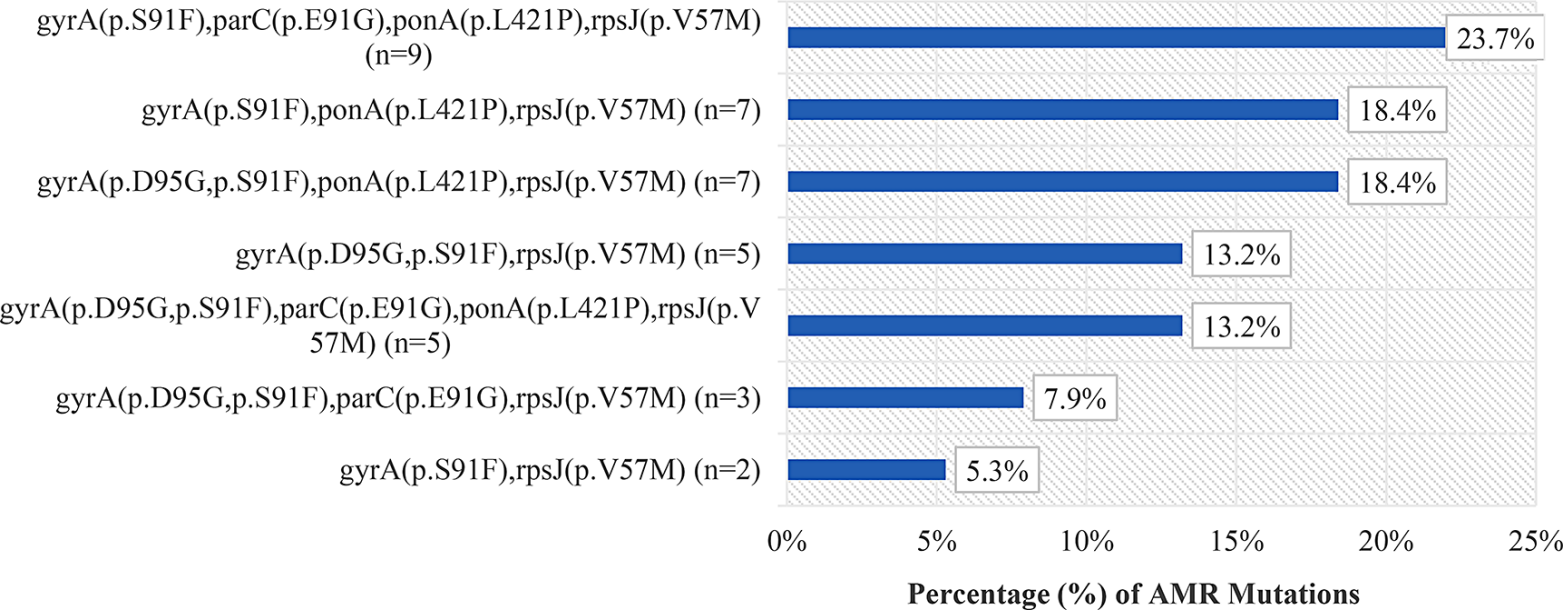
Frequency and percentage of AMR mutations from 38 *N. gonorrhoeae* isolates

The most prevalent combination of mutations of the isolates 23.7% (9/38) was *gyrA* (S91F), *parC* (E91G), *ponA* (L421) and *rpsJ* (V57M) followed by 18.4% (7/38) in *gyrA* (S91F), *ponA* (L421P), *rpsJ* (V57M), and 18.4% (7/38) in *gyrA* (D95G, S91F), *ponA* (L421P), and *rpsJ* (V57M). The combination of mutations 13.2% (5/38) *gyrA* (D95G, S91F) and *rpsJ* (V57M), and 13.2% (5/38) in the combination of *gyrA* (D95G, S91F), parC (E91F), *ponA* (L421P) and *rpsJ* (V57M) were detected. All isolates showed mutations in *gyrA* and *rpsJ* with S91F and V57M substitutions respectively (Fig. 3).

The isolates with MLST ST^7363^, ST^1921^, ST^1582^, ST^1588^, ST^1596^, ST^11181^, and ST^11750^ were associated with ciprofloxacin, penicillin and tetracycline resistance with a combination of *blaTEM-1B* and *tetM* AMR genetic determinants. Phenotypic resistance to tetracycline was detected in isolates carrying *tetM* and *rpsJ* genes while 2.6% of the isolates each exhibited AMR phenotype to cefixime and azithromycin (Table 1). The G45A deletion in *mtrR* promoter was identified in 10.6% (4/38) of the isolates. All the isolates had non-mosaic *penA* alleles which were associated with susceptibility to ceftriaxone. The identical F504L type II non-mosaic *penA* allele: 2.002 penicillin MIC 0.5 µg/mL was detected in 66% (25/38), and Type XIX non-mosaic *penA* allele: 19.001 penicillin MIC 0.5 µg/mL was detected in 34% (13/38) of the isolates.

**Table 1:** Phenotypic and genotypic characterization of isolates in MLST clusters (*n* = 38)

MLST	AMR Genotype	AMR mutation	AMR Phenotype
ST7363	blaTEM-1B,tet(M)	gyrA(p.D95G,p.S91F),parC(p.E91G),ponA(p.L421P),rpsJ(p.V57M)	CIP, PEN, TET
ST1921	blaTEM-1B,tet(M)	gyrA(p.S91F),parC(p.E91G),ponA(p.L421P),rpsJ(p.V57M	CIP, PEN, TET
ST1582	tet(M), ND	gyrA(p.D95G,p.S91F),ponA(p.L421P),rpsJ(p.V57M)	CIP, PEN, TET
ST1583	blaTEM-1B,tet(M)	gyrA(p.S91F),ponA(p.L421P),rpsJ(p.V57M)	CIP, PEN, TET, AZI
ST1588	blaTEM-1B,erm(T),tet(L) tet(M)	gyrA(p.S91F),parC(p.E91G), ponA(p.L421P), rpsJ(p.V57M)	CIP, PEN, TET, CFX
Novel	blaTEM-1B,tet(M)	gyrA(p.D95G,p.S91F),rpsJ(p.V57M)	CIP, PEN, TET
ST1596	blaTEM-1B,tet(M)	gyrA(p.S91F),ponA(p.L421P),rpsJ(p.V57M)	CIP, PEN, TET,
ST11181	blaTEM-1B,tet(M)	gyrA(p.D95G,p.S91F),rpsJ(p.V57M)	CIP, PEN, TET
ST11750	tet(M)	gyrA(p.D95G,p.S91F),parC(p.E91G),rpsJ(p.V57M)	CIP, PEN, TET
ST11241	tet(M)	gyrA(p.S91F),rpsJ(p.V57M)	CIP, PEN, TET

ND-Not detected, CIP-Ciprofloxacin, PEN-Penicillin, TET-Tetracycline, AZI-Azithromycin, CFX- Cefixime

**Fig. 4:**
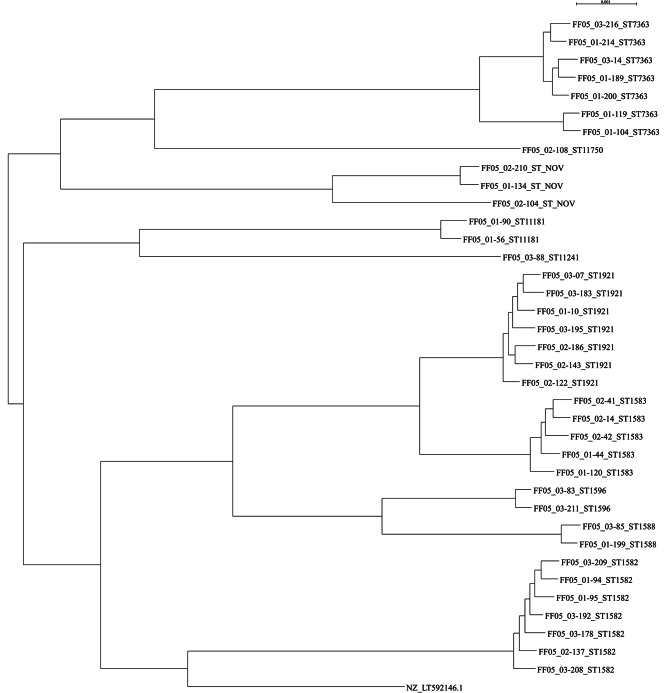
MLST phylogenetic tree of 37 *Neisseria gonorrhoeae* isolates with WHO O reference strain with GenBank accession number NZ_LT592146.1 in Lusaka, Zambia. Symbol key: NOV = Novel sequence types. The scale bar represents the estimated evolutionary divergence of the isolates

The multiple sequence alignment CCphylotree was generated using the reference strain WHO O with GenBank accession number NZ_LT592146/GCF_900087625 with MLST sequence type ST^1902^ because the majority of the isolates were closest to that reference strain. The multiple sequence alignments included 160 reference genomes for *N. gonorrhoeae* at assembly levels “Complete Genome” and “Chromosome”, retrieved on 17 Oct 2022. The isolates clustered amongst themselves rather than around the reference strain (Fig. 4). The clades showed inter-clade distances of 5000 SNPs.

## Discussion

The AMR of *N. gonorrhoeae* has been on the increase and is considered a public health challenge in various regions around the world [7]. Nowadays, WGS has been used widely in the typing and monitoring of resistant strains of *N. gonorrhoeae* [53]. Data presented in this study showed genetically diverse *N. gonorrhoeae* having 10 different MLSTs, which includes novel ST^17026^ identified in 8% (3/38) of the isolates. The three isolates with novel ST shared a single MLST profile with a novel combination of abcZ:109 adk:39 aroE:67 fumC:771 gdh:148 pdhC:71 pgm:65 [54]. The substantial number of the novelty of the isolates corresponds with findings observed in South Africa [55]. The ST^7363^, ST^1921^, and ST^1582^ representing 55% (21/38) were the most circulating sequence types in Lusaka, Zambia, and highly resistant to ciprofloxacin, penicillin, and tetracycline [33]. The combinations of amino substitutions in the *gyrA* (S91F, D95G) and *parC* (E91G) were associated with resistance to ciprofloxacin. The study findings are in agreement with studies in Tanzania and South Africa where 70% of the isolates with MLST ST^7363^ and ST^1901^ were resistant to ciprofloxacin, penicillin, and tetracycline [59, 60]. Penicillinase-producing *N. gonorrhoeae* (PPNG) strains 84.2% (32/38) were detected in all the STs, and 2.6% (1/38) of the isolates had reduced susceptibility to cefixime with MIC 0.75 µg/mL (Additional file 1: Table S1). The PPNG isolates are highly prevalent and the global concern is that the *bla*_TEM−1_ gene encoding _TEM−1_ β-lactamase requires few specific single nucleotide polymorphisms (SNPs) to evolve into a gene encoding an extended-spectrum β-lactamase (ESBL) which could degrade all cephalosporins including ceftriaxone [61, 62]. All isolates harbored the GGI which is a type IV secretion system (T4SS) implicated in AMR to multiple antimicrobials [63]. Mutations within the *mtrR* gene are usually found amongst multidrug-resistant isolates of *N. gonorrhoeae* and are associated with the outflow of antimicrobials from the bacterial cell [64]. Furthermore, 2.6% of the isolates exhibited resistance to azithromycin, and all the isolates were susceptible to ceftriaxone [33]. The rise in the number of reports on treatment failures with 3GS demands urgent development of new antimicrobials for the treatment of gonorrhea and increased AMR surveillance remains vital to the prevention and control of gonorrhea worldwide [65–67].

## Conclusion

The genomic analysis of the study showed a remarkable genetic diversity of *N. gonorrhoeae* with *bla*_TEM−1_, *tetM, ponA, gyrA*, and *parC* genes associated with high resistance to penicillin, tetracycline and ciprofloxacin demanding for revision of the Zambian standard treatment guidelines. The detection of *N. gonorrhoeae* resistant to azithromycin demands improved antimicrobial stewardship to prevent an epidemic of untreatable gonorrhea in Zambia.

### Limitation of the study

The isolates studied were only from hospitals in Lusaka and might not be representative of other settings in Zambia.

### Electronic supplementary material

Below is the link to the electronic supplementary material.

Supplementary Material 1

## Data Availability

The genomic data generated and analyzed in this study have been deposited in the European Nucleotide Archive (ENA) at EMBL-EBI under accession number PRJEB68050 (https://www.ebi.ac.uk/ena/browser/view/PRJEB68050). Genomes with novel ST17026 have additionally been deposited in the Neisseria spp. database at PubMLST, accessions 123150-2 (https://pubmlst.org/bigsdb?page=info&db=pubmlst_neisseria_isolates&id=123150).
