# Supplementary material for: Antimicrobial resistance and heterogeneity of Neisseria gonorrhoeae isolated from patients attending sexually transmitted infection clinics in Lusaka, Zambia
Source: BMC Genomics. 2024 Mar 18;25:290. doi: 10.1186/s12864-024-10155-y (PMC10949682; doi:10.1186/s12864-024-10155-y)
Supplement: Supplementary file 1 — Supplementary Material 1 [file 12864_2024_10155_MOESM1_ESM.docx]

**Supplementary Information**

**Tables S1**: *In vitro* antimicrobial Profile by MIC (µg/mL) using E- test method (n = 38)

|  | Number of Isolate (%) | | |  | MIC (µg/mL) | | |
| --- | --- | --- | --- | --- | --- | --- | --- |
| Antimicrobial Agent | S | I | R |  | Range | MIC_50_ | MIC_90_ |
| Ciprofloxacin | 0 (0) | 0(0) | 38 (100) |  | 1-32 | 8 | 32 |
| Ceftriaxone | 38 (100) | 0(0) | 0 (0) |  | 0.016-0.125 | 0.016 | 0.132 |
| Spectinomycin | 38 (0) | 0(0) | 0(0) |  | 0.19-8 | 2 | 8 |
| Cefixime | 37(97.4) | 0(0) | 1 (2.6) |  | 0.016-0.75 | 0.016 | 0.125 |
| Azithromycin | 37(97.4) | 0(0) | 1(2.6) |  | 0.016-2 | 0.125 | 0.25 |
| Penicillin G | 0 (0) | 0(0) | 38 (100) |  | 4-32 | 32 | 32 |
| Tetracycline | 0 (0) | 0(0) | 38(100) |  | 4-64 | 4 | 8 |

*S -* Susceptible,  *I* - Intermediate, *R* - Resistant, MIC_50_ and MIC_90_: MIC value to inhibit 50% and 90 % of the strains tested respectively
